# Genetic background determines severity of *Loxl1*-mediated systemic and ocular elastosis in mice

**DOI:** 10.1242/dmm.050392

**Published:** 2023-11-13

**Authors:** Maria F. Suarez, Heather M. Schmitt, Megan S. Kuhn, TeddiJo Watkins, Kristyn M. Hake, Tara Weisz, Edward J. Flynn, Michael H. Elliott, Michael A. Hauser, W. Daniel Stamer

**Affiliations:** ^1^ Department of Ophthalmology, Duke University, Durham, NC 27705, USA; ^2^ Duke Molecular Physiology Institute, Duke University, Durham, NC 27701, USA; ^3^ Department of Ophthalmology, Dean McGee Eye Institute, University of Oklahoma Health Sciences Center, Oklahoma City, OK 73104, USA; ^4^ Department of Medicine, Duke University, Durham, NC 27710, USA

**Keywords:** Trabecular meshwork, Lysyl oxidase, Pseudoexfoliation syndrome, Glaucoma, Mechanical stress

## Abstract

Pseudoexfoliation syndrome (PEX) is a systemic, age-related disorder characterized by elastosis and extracellular matrix deposits. Its most significant ocular manifestation is an aggressive form of glaucoma associated with variants in the gene encoding lysyl oxidase-like 1 (*LOXL1*). Depending upon the population, variants in *LOXL1* can impart risk or protection for PEX, suggesting the importance of genetic context. As LOXL1 protein levels are lower and the degree of elastosis is higher in people with PEX, we studied *Loxl1*-deficient mice on three different genetic backgrounds: C57BL/6 (BL/6), 129S×C57BL/6 (50/50) and 129S. Early onset and high prevalence of spontaneous pelvic organ prolapse in BL/6 *Loxl1^−/−^* mice necessitated the study of mice that were <2 months old. Similar to pelvic organ prolapse, most elastosis endpoints were the most severe in BL/6 *Loxl1^−/−^* mice, including skin laxity, pulmonary tropoelastin accumulation, expansion of Schlemm's canal and dilation of intrascleral veins. Interestingly, intraocular pressure was elevated in 50/50 *Loxl1^−/−^* mice, depressed in BL/6 *Loxl1^−/−^* mice and unchanged in 129S *Loxl1^−/−^* mice compared to that of control littermates. Overall, the 129S background was protective against most elastosis phenotypes studied. Thus, repair of elastin-containing tissues is impacted by the abundance of LOXL1 and genetic context in young animals.

## Teaser

**Summary:** Depending upon genetic context and age, loss of the repair enzyme lysyl oxidase-like 1 in mice places tissues subject to repetitive cycles of mechanical stress at risk for elastosis.

## INTRODUCTION

Pseudoexfoliation syndrome (PEX) is an age-related disorder that affects many ocular and non-ocular tissues. Among the ocular manifestations of PEX, cataract and glaucoma are the most significant clinically (Ritch, 2018), with PEX considered as the most common identifiable cause of open-angle glaucoma worldwide (Ritch, 1994; Schlötzer-Schrehardt, 2011; Desai and Lee, 2008). PEX glaucoma (PEXG) is characterized by a pronounced asymmetry in ocular extracellular matrix (ECM) fibrillary material, ocular hypertension, resistance to drug therapy, higher rates of surgery and rapid progression to blindness (Aboobakar et al., 2017; Ritch, 1994; Desai and Lee, 2008; Mitchell et al., 1999).

The abnormal deposition of fibrillar ECM material in ocular and extra-ocular tissues (Aboobakar et al., 2017; Schlotzer-Schrehardt et al., 2008; Schlotzer-Schrehardt, 2009; Schlotzer-Schrehardt and Naumann, 2006) is found in all the structures of the anterior segment of the eye, the conjunctiva and the orbital structures, and also in the heart, kidney, liver, skin, gallbladder and cerebral meninges, emphasizing the systemic nature of the disease (Schlötzer-Schrehardt et al., 1992; Streeten et al., 1992; Ritch, 2016; Ritch and Schlötzer-Schrehardt, 2001; Schlotzer-Schrehardt and Naumann, 2006). Many components of PEX extracellular deposits have been identified, including basement membrane proteins such as laminin and fibronectin (FN1), and a number of proteins from the elastic fiber system including fibrillin-1 (FBN1), latent-transforming growth factor-binding proteins (LTBP-1, LTBP-2, LTBP-3 and LTBP-4), fibulins [FBLN-2, FBLN-4 (also known as EFEMP4) and FBLN-6 (or HMCN1)], vitronectin (VTN), tropoelastin and elastin (ELN), clusterin (CLU), apolipoprotein E (ApoE) and lysyl oxidase (LOX)-like 1 (LOXL1) (Aboobakar et al., 2017; Zenkel and Schlötzer-Schrehardt, 2014a; Challa and Johnson, 2018; Ritch and Schlötzer-Schrehardt, 2001).

Elastic fibers are long-lived constituents of the ECM, which provides elasticity and resilience to organs and tissues subjected to mechanical stress, including skin, lungs, blood vessels, ligaments and ocular tissues (Heinz, 2021; Schmelzer and Duca, 2022). Similarly, the trabecular meshwork (TM) of the ocular conventional outflow pathway experiences repetitive mechanical stress (Xin et al., 2018). Mechanical deformation of the TM is reinforced by a cribriform elastin plexus that extends from the ciliary muscle tips to the inner wall of Schlemm's canal (SC), the primary drainage vessel for aqueous humor (Lütjen-Drecoll et al., 1981; Hann and Fautsch, 2011; Overby et al., 2014). Such support by the cribriform elastin plexus enables the juxtacanalicular (JCT) region of the TM to expand and recoil during these repetitive intraocular pressure (IOP) fluctuations (Keller and Acott, 2013). In healthy eyes, these repetitive mechanical stimuli are monitored by TM and SC cells to regulate outflow resistance and IOP homeostasis (Acott et al., 2014). In PEXG eyes, this feedback loop might be disrupted due to tissue laxity and/or fibrillar deposition (elastosis) of the JCT region, which is coincident with elevated IOP. Prolonged exposure of retinal ganglion cell axons at the optic nerve head to elevated IOP results in cellular apoptosis and blindness (Schlotzer-Schrehardt and Naumann, 2006; Naumann et al., 1998; Gelman et al., 2010).

The formation and repair of elastin fibers requires the action of LOX enzymes (Doué et al., 2021; Shin and Yanagisawa, 2019). The LOX family consists of five members, LOX and LOX-like (LOXL) 1-4, which crosslink collagen and elastin (Smith-Mungo and Kagan, 1998; Vallet and Ricard-Blum, 2019). LOXL1, a major component of PEX deposits, has a special role in the repair of elastin in tissues such as the TM that experience repetitive and traumatic mechanical strain (Sethi et al., 2011; Wordinger and Clark, 2014). LOXL1, but not the related LOX family members that specifically localize to elastogenesis sites guiding elastin deposition onto the existing polymer (Vrhovski and Weiss, 1998; Liu et al., 2004). Importantly, variants in the *LOXL1* gene are associated with high risk for ocular hypertension and PEXG (Thorleifsson et al., 2007; Aung et al., 2017). Variants associated with risk in some populations are protective in others. This allelic reversal complicates the genetic landscape and the interpretation of the role of potential functional variants (Aboobakar et al., 2017). In terms of protein levels, LOXL1 expression is altered with disease progression, being elevated at early stages and downregulated at late stages of the disease (Schlotzer-Schrehardt et al., 2008; Schlotzer-Schrehardt, 2009; Zenkel and Schlötzer-Schrehardt, 2014a).

Due to decreased LOXL1 expression with disease progression, *Loxl1*-deficient mice have been used as a model for PEX. For example, *Loxl1* knockout mice (*Loxl1^−/−^* on a mixed C57BL/6J and 129S1/SvImJ background) have abnormal elastic fiber appearance in the uterine tract postpartum, resulting in pelvic organ prolapse, enlarged alveoli in lungs, increased skin laxity and vascular abnormalities with tropoelastin accumulation (Liu et al., 2004). In the eye, *Loxl1^−/−^* mice have lens abnormalities, blood-aqueous barrier disruption and elastic tissue dysfunction in the conventional outflow pathway, which leads to ocular hypertension (Wiggs et al., 2014; Li et al., 2020). To model the genetic context of LOXL1-mediated elastosis, we crossed *Loxl1^−/−^* mice of mixed background onto two different backgrounds: C57BL/6J and 129S1/SvImJ. Here, we describe the dramatic differences in ocular and non-ocular elastic tissue phenotypes associated with *Loxl1^−/−^* mice in three different genetic backgrounds: C57BL/6 (BL/6), 129S×C57BL/6 (50/50) and 129S.

## RESULTS

### Young BL/6 *Loxl1^−/−^* mice have increased probability of prolapse, reduced weight and delayed elastic skin recovery

**Fig. 1.**
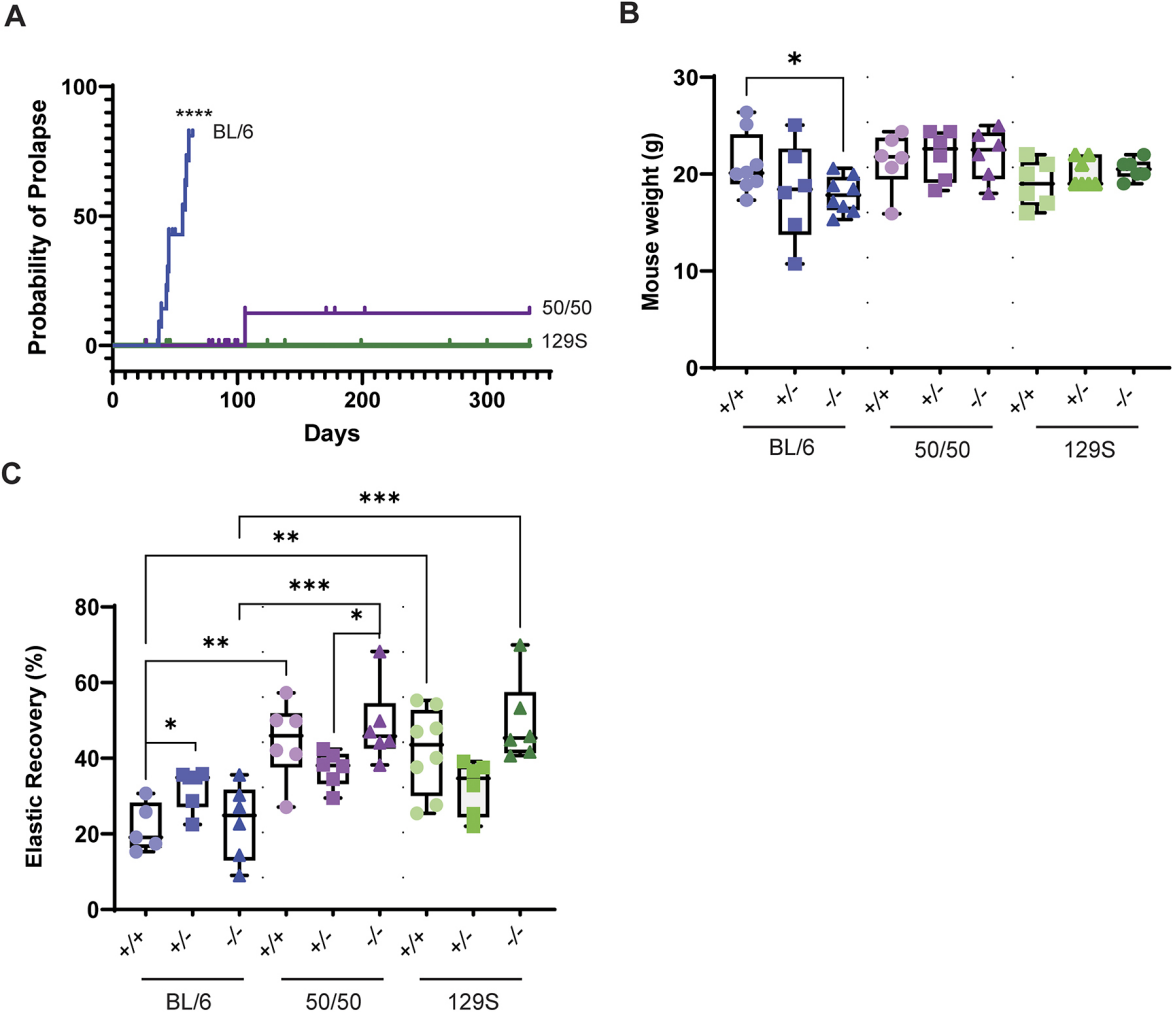
***Loxl1* deficiency and genetic background affects probability of prolapse, weight gain and skin laxity.** (A) The probability of spontaneous organ prolapse increased to 81% at postnatal day 60 in BL/6 *Loxl1^−/−^* mice (*n*=16) and to 12.5% at postnatal day 106 in 50/50 *Loxl1^−/−^* mice (*n*=25), and remained unchanged until the last day of follow up (postnatal day 334) for 129S *Loxl1^−/−^* mice (0%, *n*=16). BL/6 *Loxl1^−/−^* versus 50/50 *Loxl1^−/−^* and BL/6 *Loxl1^−/−^* versus 129S *Loxl1^−/−^*, ****P<0.0001. (B) Weight was monitored in 2-month-old *Loxl1^+/+^*, *Loxl1^+/−^* and *Loxl1^−^*^/*−*^ mice from the three different strains (BL/6, *n*=6-8; 50/50, *n*=6 for all genotypes; 129S, *n*=6-7). *P<0.05. (C) Skin recovery displacement after applying a suction force was quantified in all three genotypes from BL/6 (*n*=5-6), 50/50 (*n*=6) and 129S (*n*=6-8) background mice (*P<0.05; **P<0.01; ***P<0.001). For B,C, boxes show the 25-75th percentiles, whiskers show the minimum and maximum, and the median is marked with a line. For A-C, ordinary one-way ANOVA followed by Tukey's multiple comparisons test was used for statistical analysis.

In addition to both genders suffering from increased rectal prolapse with age, it was reported that *Loxl1^−/−^* female mice on a mixed background display pelvic organ prolapse 2 days postpartum, with retraction at day 14; but with permanent damage to the pelvic floor (Liu et al., 2004; Li et al., 2020). We backcrossed the mixed strain (Li et al., 2020) onto C57BL/6J or 129S1/SvImJ for six generations (N6) to test the effect of a defined background on elastosis phenotypes. For comparisons to the ‘mixed’ strain, we also crossed the N6 C57BL/6 *Loxl1^−/−^* mice into the 129S1/SvImJ background for one generation, giving a ‘50/50’ background. We found that the N6 BL/6 mice were 98.6% BL/6 by DartMouse^TM^ genotyping services and presented with pelvic organ prolapse by 2 months of age, whereas the 50/50 and 129S *Loxl1^−/−^* mice did not. The probability of spontaneous organ prolapse in the BL/6 *Loxl1^−/−^* mice was 81% at postnatal day 60, whereas this probability was 0% for both 50/50 *Loxl1^−/−^* and 129S *Loxl1^−/−^* mice at the same age. We continued to follow the 50/50 and 129S mice and found that at postnatal day 106, the probability of prolapse for the 50/50 strain increased to 12.5%, and remained unchanged until postnatal day 334. For 129S mice, the probability of prolapse did not change (0%) over >300 days of observation (Fig. 1A).

The early onset and high prevalence of spontaneous organ prolapse of *Loxl1^−/−^* mice on the BL/6 background constrained our direct comparative studies between genotypes to 2-month-old mice. In accordance with the deteriorated health condition due to the organ prolapse, the weight of BL/6 *Loxl1^−/−^* mice was significantly reduced compared to that of their *Loxl1^+/+^* littermates (*P*=0.034), whereas the weights of 50/50 and 129S mice were not different among the *Loxl1^+/+^*, *Loxl1^+/−^* and *Loxl1^−/−^* genotypes (*P*>0.05) (Fig. 1B).

Previously, 4-month-old *Loxl1^−/−^* mice on the mixed background (with unknown content of BL/6 versus 129S) presented with qualitative increased skin laxity (Liu et al., 2004). To quantify these changes in mice with defined backgrounds (*n*=5-8 mice per genotype, per genetic background), we used a cutometer to measure the recovery of skin displaced by a consistent force of applied suction on 2-month-old mice. For mice with the BL/6 genetic background, skin elastic recovery was significantly higher in *Loxl1^+/−^* mice compared to that in *Loxl1^+/+^* littermates (*P*=0.02), but skin elastic recovery was significantly reduced in *Loxl1^+/+^* and *Loxl1^−/−^* mice on the BL/6 background compared to that in the *Loxl1^+/+^* and *Loxl1^−/−^* mice on the other two backgrounds (BL/6 *Loxl1^+/+^* versus 50/50 *Loxl1^+/+^*, *P*=0.044; BL/6 *Loxl1^+/+^* versus 129S *Loxl1^+/+^*, *P*=0.01; BL/6 *Loxl1^−/−^* versus 50/50 *Loxl1^−/−^*, *P*=0.0004; and BL/6 *Loxl1^−/−^* versus 129S *Loxl1^−/−^*, *P*=0.0003) (Fig. 1C). In general, the BL/6 content of mice reduced elastic recovery, regardless of *Loxl1* expression.

### Alveolar morphology and tropoelastin expression is altered in young *Loxl1^−/−^* mice

**Fig. 2.**
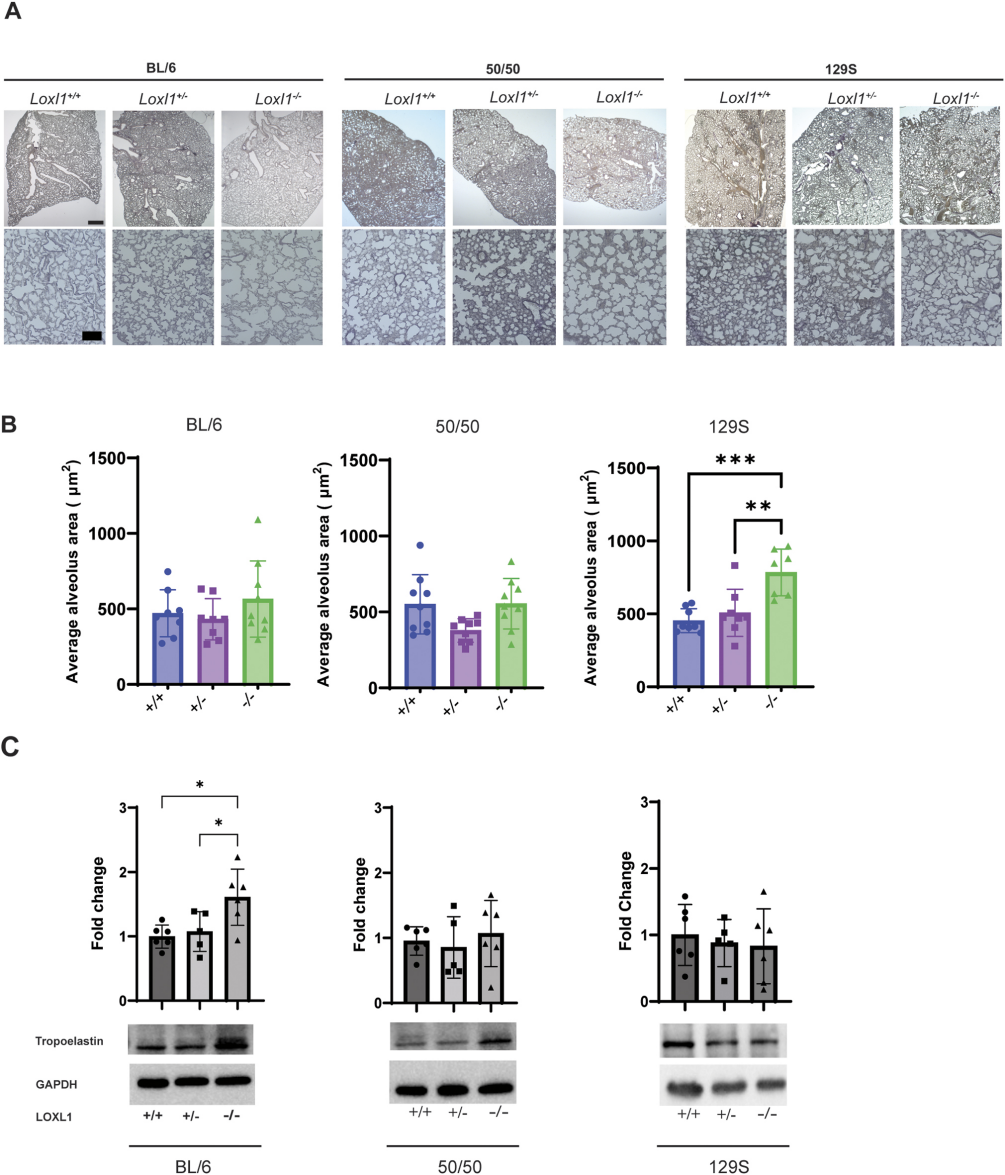
***Loxl1* deficiency alters morphology and tropoelastin expression in young mouse lungs.** (A) Representative images of Weigert's Resorcin-Fuchsin-stained sagittal lung sections showing expanded alveolar spaces in 2-month-old *Loxl1^−/−^* BL/6, 50/50 and 129S mice. Scale bars: 100 μm (upper panels); 20 μm (lower panels). (B) The average alveolus area was quantified from five images from four mice/genotype/background at 20× magnification using particle analysis in Fiji. (C) Tropoelastin expression was determined by immunoblotting in lung tissue homogenates from BL/6, 50/50 and 129S mice (*n*=5-6 mice/genotype/background) and normalized to GAPDH expression. Bars show the mean±s.d. For B and C, ordinary one-way ANOVA followed by Tukey's multiple comparisons test was used for statistical analysis. *P<0.05; **P<0.01; ***P<0.001.

Elastin provides elastic recoil to the alveoli in the lungs, enabling proper function during breathing (Mecham, 2018; Luo et al., 2018). Enlarged alveolar spaces have been described in lung sections from 3- to 5-month-old *Loxl1^−/−^* mice on the mixed background, compatible with an emphysematous change (Liu et al., 2004). Here, Weigert's Resorcin-Fuchsin-stained lung sections were imaged and alveolar space areas calculated. Visual inspection revealed modestly enlarged alveoli for the *Loxl1^−/−^* mice on all three different backgrounds (Fig. 2A). However, image quantification showed that alveolar spaces were only significantly different for the 129S *Loxl1* null mice compared to those of both *Loxl1^+/+^* and *Loxl1^+/−^* littermates (*P*=0.0005 and 0.003, respectively) (Fig. 2B). No significant differences in alveolar spaces were detected in *Loxl1^−/−^* mice compared to those of their *Loxl1^+/+^* or *Loxl1^+/−^* littermates for either the BL/6 (*P*=0.69 and 0.42, respectively) or 50/50 (*P*>0.99 and *P*=0.07, respectively) backgrounds.

Accumulation of tropoelastin (elastin monomer) in lung and other tissues from 5- to 10-month-old mixed-strain *Loxl1^−/−^* mice was previously reported by western blotting (Liu et al., 2004; Wiggs et al., 2014). Similarly, immunoblots of lung tissue samples in this study showed a significant increase in tropoelastin expression in 2-month-old BL/6 *Loxl1^−/−^* mice compared to that of their *Loxl1^+/+^* (*P*=0.016) and *Loxl1^+/−^* (*P*=0.044) littermates (Fig. 2C). No significant changes were detected in the 2-month-old *Loxl1^−/−^* mice from the other two backgrounds (Fig. 2C).

### IOP regulation is paradoxically affected by genetic background in *Loxl1^−/−^* mice

**Fig. 3.**
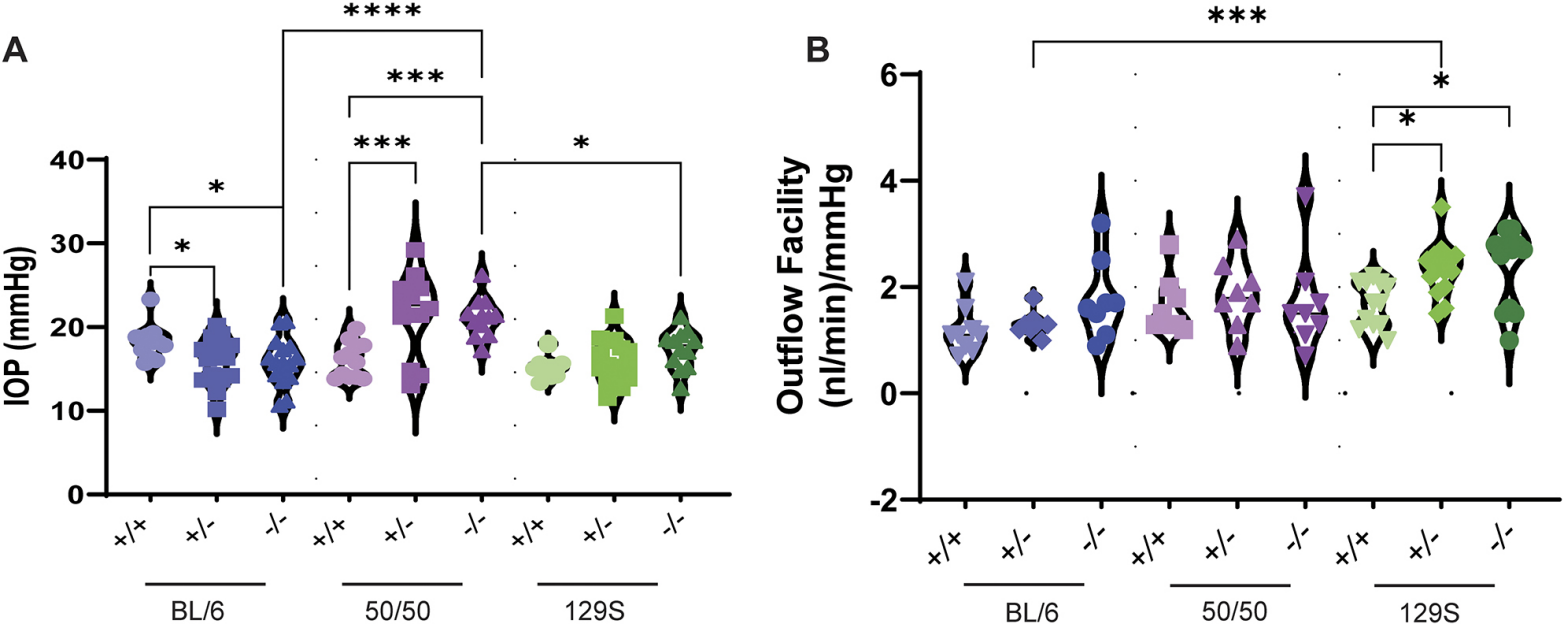
**IOP and outflow facility are differentially affected in young *Loxl1* mutant mice on three different genetic backgrounds.** (A) Two-month-old BL/6 *Loxl1^+/−^* and *Loxl1^−/−^* mice displayed decreased IOP (*n*=6-8 mice, 12-16 eyes, *P<0.05), whereas 2-month-old 50/50 *Loxl1^+/−^* and *Loxl1^−/−^* mice presented with increased IOP (*n*=6 mice, 12 eyes, ***P<0.001). Two-month-old mice on the 129S background showed a trend of increasing IOP (*n*=6-8 mice, 12-16 eyes, *P*>0.05) (B) The outflow facility was unchanged except in *Loxl1^+/−^* and *Loxl1^−/−^* mice on the 129S background (*n*=6-7 mice, 12-14 eyes, *P<0.05). BL/6: *n*=4-5 mice, 8-10 eyes; 50/50: *n*=4 mice, 8 eyes. Violin plots show the full distribution of all data points acquired in A,B, and ordinary one-way ANOVA followed by Holm-Šídák's multiple comparisons test was used for statistical analysis.

Previously, we observed that young (3-month-old) *Loxl1^−/−^* mice and middle-aged (8- to 12-month-old) *Loxl1^−/−^* mice on the unknown ‘mixed’ background had significantly elevated IOPs compared to those of their age-matched *Loxl1^+/+^* littermates, with no differences between *Loxl1^+/−^* and *Loxl1^+/+^* mice (Li et al., 2020). In this study, we observed similar results, where IOPs in 2-month-old 50/50 mice were significantly increased for both the *Loxl1^+/−^* and *Loxl1^−/−^* genotypes (21.50±5.03 mmHg, *n*=6, *P*=0.0001 and 21.13±2.33 mmHg, *n*=6, *P*=0.0004, respectively) versus those of their *Loxl1^+/+^* littermates (16.07±2.09 mmHg, *n*=6) (Fig. 3A). In contrast, IOPs were significantly reduced in *Loxl1^+/−^* and *Loxl1^−/−^* mice on a BL/6 background (15.98±2.73 mmHg, *n*=8, *P*=0.02 and 15.79±2.79 mmHg, *n*=8, *P*=0.01, respectively) compared to those of their *Loxl1^+/+^* littermates (18.24±2.02 mmHg, *n*=6). IOPs in *Loxl1^−/−^* mice on a 129S background were not different from those of their *Loxl1^+/+^* littermates (17.32±2.34 mmHg, *n*=6 versus 15.35±1.42 mmHg, *n*=6; *P*=0.77) (Fig. 3A). When comparing *Loxl1^−/−^* mice across the different backgrounds, IOP in 50/50 mice was significantly elevated versus IOPs in BL/6 mice (*P*<0.0001) and 129S mice (*P*=0.024). Taken together, the expression levels of *Loxl1* affect IOP regulation differently depending upon genetic context.

To determine whether IOP variations were due to biomechanical changes in the cornea caused by the lack of *Loxl1* in our recent study, we monitored corneal thickness and tested readings from our rebound tonometer to direct measurements by anterior chamber cannulation and observed no differences (Li et al., 2020). To continue monitoring for possible changes in the cornea due to *Loxl1* deficiency in the present study, we measured corneal thickness in histological sections from anterior segments stained with Weigert's Resorcin-Fuchsin stain. Similar to the previous study, we found that corneal thickness was not altered in *Loxl1^−/−^* mice compared to that in their respective *Loxl1^+/+^* littermates (Fig. S4).

### Outflow facility is enhanced in young *Loxl1^−/−^* mice on the three different backgrounds

To determine whether changes in IOP were due to impaired outflow function, we measured outflow facilities (inverse of outflow resistances) (Sherwood et al., 2016). In general, outflow facility increased with decreasing expression of *Loxl1*, regardless of genetic background. However, outflow was not significantly different in *Loxl1^−/−^* mice compared to that in *Loxl1^+/+^* littermates with either the BL/6 or 50/50 background (*P*=0.77 and >0.99, respectively). In contrast, outflow facility in *Loxl1^−/−^* mice on the 129S background was significantly increased compared to that of their *Loxl1^+/+^* littermates (2.35±0.21 nl/min/mmHg versus 1.67±0.12 nl/min/mmHg; *P*=0.013). Additionally, comparison between the different backgrounds showed that facilities in 129S *Loxl1^+/−^* mice were significantly increased with respect to those of BL/6 *Loxl1^+/−^* mice (2.33±0.50 nl/min/mmHg versus 1.31±0.23 nl/min/mmHg; *P*=0.019) (Fig. 3B).

### SC lumen is enlarged in young *Loxl1^−/−^* mice

**Fig. 4.**
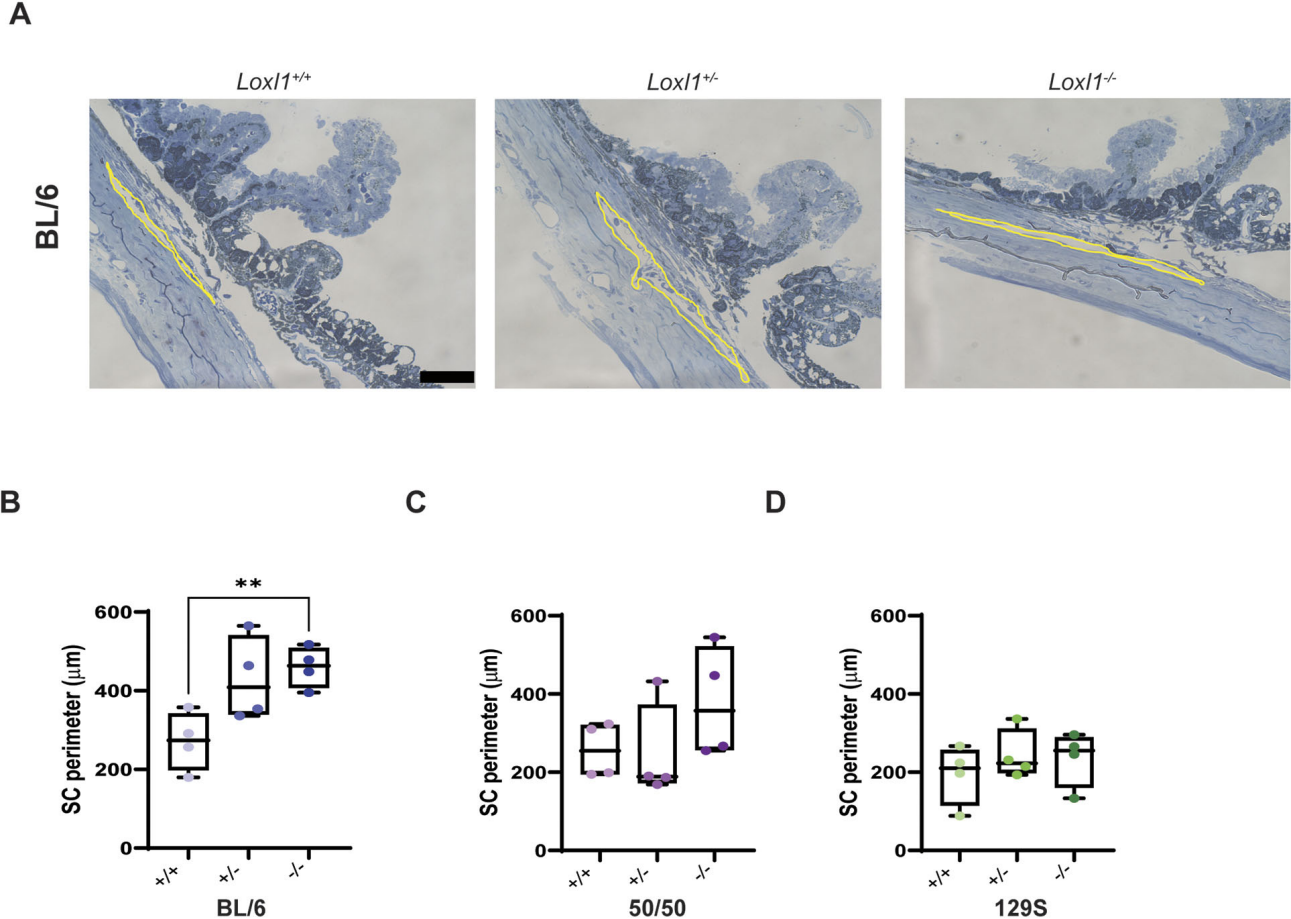
***Loxl1* deficiency in mice containing BL/6 causes enlargement of Schlemm's canal lumen.** (A) Representative images of the outflow tract from sagittal semi-thin sections stained with Methylene Blue in BL/6 *Loxl1^+/+^*, *Loxl1^+/−^* and *Loxl1^−/−^* mice. The Schlemm's canal (SC) perimeter is highlighted in yellow. Scale bar: 20 µm. (B) Quantitative analysis of SC perimeter revealed an enlarged SC in BL/6 *Loxl1^+/−^* and *Loxl1^−/−^* mice and in 50/50 *Loxl1^−/−^* mice (*n*=4 mice, averaging three cross-sectional images/eye; **P<0.01). Boxes show the 25-75th percentiles, whiskers show the minimum and maximum, and the median is marked with a line. Ordinary one-way ANOVA followed by Dunnett's multiple comparisons test was used for statistical analysis.

The conventional outflow pathway tissue is rich in elastin (Hann and Fautsch, 2011; Overby et al., 2014), which localizes with LOXL1 expression (Schlotzer-Schrehardt et al., 2008). Li et al. (2020) reported that older (8- to 12-month-old) *Loxl1^−/−^* mice on the ‘mixed’ background presented with significant enlargement of the SC lumen. Here, measurements of SC in sagittal semi-thin sections (Fig. 4A) showed that its perimeter is significantly enlarged in BL/6 *Loxl1^−/−^* mice compared to that in their *Loxl1^+/+^* littermates (459.8±51.6 µm versus 271.4±73.97 µm, respectively; *P*=0.008, *n*=4 for each genotype) (Fig. 4B), but there were no significant differences in SC perimeter between *Loxl1^−/−^* and *Loxl1^+/−^* BL/6 mice. Similar to our previous study, SC perimeter in young 50/50 *Loxl1^−/−^* mice exhibited a trend towards enlargement, compared to that in the *Loxl1^+/+^* and *Loxl1^+/−^* mice, but this change was not significant (*n*=4 for each genotype, *P*=0.34 and 0.28, respectively) (Fig. 4C), whereas 129S mice did not show changes in SC perimeter among genotypes (*n*=4 for each genotype, *P*>0.05) (Fig. 4D). Additionally, SC in BL/6 *Loxl1^−/−^* mice was significantly distended compared to 129S *Loxl1^−/−^* mice (459.8±51.62 µm versus 234.9±70.89 µm, *P*=0.0098).

### Distal veins are dilated in young BL/6 * Loxl1^−/−^* mice

**Fig. 5.**
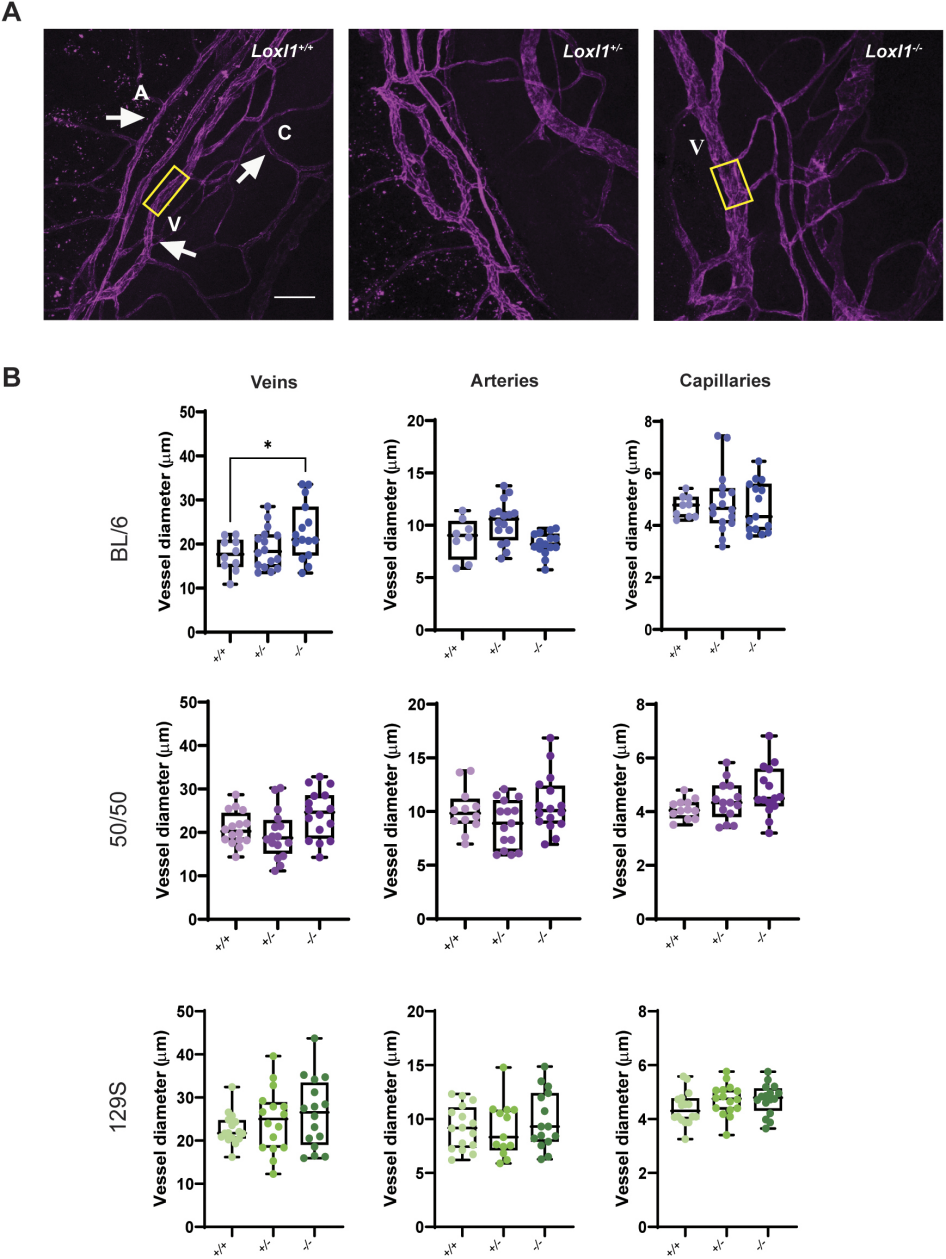
**Young BL/6 *Loxl1^−/−^* mice have dilated intrascleral veins.** (A) Representative images of anterior chamber flat mounts immunostained with anti-CD31 antibody in BL/6 *Loxl1^+/+^*, *Loxl1^+/−^* and *Loxl1^−/−^* mice. Yellow rectangles highlight the difference between distal vein diameter in *Loxl1^+/+^* versus *Loxl1^−/−^* mice. A, artery; C, capillary; V, vein. Scale bar=100 µm. (B) Vessel diameter measurements for *Loxl1^+/+^*, *Loxl1^+/−^* and *Loxl1^−/−^* mice from the three different studied backgrounds. Only BL/6 *Loxl1^−/−^* mice displayed significantly dilated distal veins but not arteries or capillaries (*n*=4 eyes/mice, 4 quadrants/eye, *P<0.05). Boxes show the 25-75th percentiles, whiskers show the minimum and maximum, and the median is marked with a line. Ordinary one-way ANOVA followed by Dunnett's multiple comparisons test was used for statistical analysis.

Distal vessels drain aqueous humor from SC into the systemic circulation and are responsible for 25-50% of total outflow resistance (McDonnell et al., 2018). Our prior study in old mice from the ‘mixed’ background showed that distal veins, but not arteries or capillaries, were significantly dilated in *Loxl1^−/−^* mice compared to those in *Loxl1^+/+^* littermates (Li et al., 2020). Here, we quantified the size of distal vessels in anterior chamber flat mounts from *Loxl1* mice of three defined backgrounds (Fig. 5A) and found that young *Loxl1^−/−^* mice on the BL/6 background had significantly dilated veins compared to those of their *Loxl1^+/+^* littermates (22.88±6.44 µm versus 17.62±3.73 µm, *P*=0.045) (Fig. 5B), without changes in arteries or capillaries. Similar to the older mice on the ‘mixed’ background that we analyzed previously, the average vein diameter for the young 50/50 *Loxl1^−/−^* mice showed a trend towards dilation, compared to that of their *Loxl1^+/+^* littermates (*P*=0.16). Interestingly, the capillaries, but not the arteries, trended to larger diameters in young 50/50 *Loxl1^−/−^* mice. Additionally, the 129S background showed an increased trend in vein diameter in *Loxl1^−/−^* mice versus that in *Loxl1^+/+^* littermates (*P*=0.31), but no changes in artery or capillary diameters.

### Pulmonary expression of Lox family members in *Loxl1*-deficient mice

**Fig. 6.**
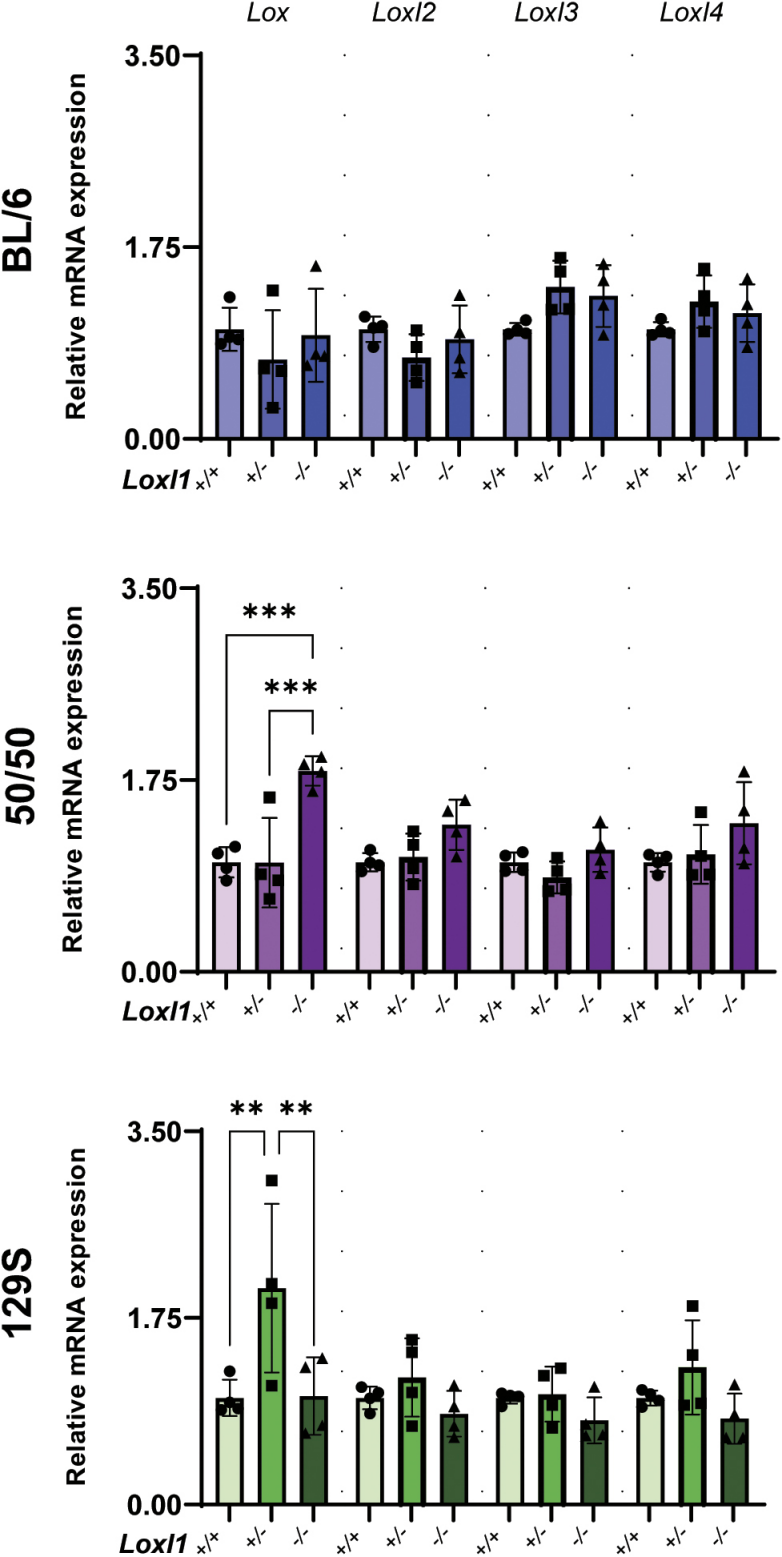
**Effects of *Loxl1* deficiency on expression of lysyl oxidase family members in mice of defined backgrounds*.*** Messenger RNA levels for *Lox*, *Loxl2*, *Loxl3* and *Loxl4* from lung tissues of *Loxl1^+/+^*, *Loxl1^+/−^* and *Loxl1^−/−^* BL/6, 50/50 and 129S mice were analyzed by qPCR (*n*=4 mice/genotype/genetic background) and normalized to *Gapdh* expression levels. Bars show the mean±s.d. Ordinary one-way ANOVA followed by Tukey's multiple comparisons test was used for statistical analysis. **P<0.01, ***P<0.001.

Previous studies that examined *Loxl1^−/−^* mice from the original ‘mixed’ strain showed that LOXL1 has a nonredundant role in elastic fiber homeostasis in adult mouse tissues (Liu et al., 2004). Here, we wanted to determine whether the paradoxical physiological changes observed in the young *Loxl1*-deficient mice from the three defined backgrounds could be related to compensation by other members of the lysyl oxidase family. To this end, we performed quantitative PCR (qPCR) in lung tissue from *Loxl1^+/+^*, *Loxl1^+/−^* and *Loxl1^−/−^* BL/6, 50/50 and 129S background mice (*n*=4 mice/genotype/background) for *Lox*, *Loxl2*, *Loxl3* and *Loxl4.* Interestingly, we found that only *Lox* was upregulated in *Loxl1^−/−^* mice on the 50/50 background (*P*=0.0004) and in *Loxl1^+/−^* mice on the 129S background (*P*=0.0055) compared to their respective *Loxl1^+/+^* littermates (Fig. 6). In contrast, we observed no alterations in *Loxl1^−/−^* or *Loxl1^+/−^* mice on the BL/6 background compared to their *Loxl1^+/+^* littermates.

### *Loxl1*-deficient mice exhibit altered ECM deposition in the inner wall of SC

Previous work described a thickening of the basal lamina and increased disorganized fibrillary deposits beneath the endothelial cells of the SC inner wall in aged *Loxl1^−/−^* mice on the ‘mixed’ background compared to those of their corresponding *Loxl1^+/−^* and *Loxl1^+/+^* littermates by light microscopy (Li et al., 2020). In the present study, qualitative analyses at the ultrastructural level showed an increased deposition in basal laminar materials under the inner wall of the SC for *Loxl1^−/−^* mice on each background (BL/6, 50/50 and 129S, *n*=3-5 sections per eye with four eyes/genotype) compared to their respective *Loxl1^+/−^* and *Loxl1^+/+^* littermates (Fig. S5). Among the genetic backgrounds, the deposited material was more abundant and continuous in young BL/6 *Loxl1^−/−^* mice compared to that in young *Loxl1^−/−^* mice from the 50/50 or 129S background. Additionally, material deposits in 129S *Loxl1^−/−^* mice appeared more discontinuous or ‘patchy’, and they were not consistent in appearance through all the analyzed sections. Although we generally observed more deposits in the JCT region of *Loxl1^−/−^* mice on the BL/6 background, the location and abundance were not consistent and thus quantification was not feasible*.* Thus, matrix changes appear to start early in these young mice and seem to be more severe with age (Li et al., 2020) and increasing amount of BL/6 genetic content.

## DISCUSSION

PEX is an aging disorder characterized by ECM dysregulation and with LOXL1, a major component of PEX deposits, largely implicated in its pathogenesis. Despite the strong association between genetic polymorphisms at the *LOXL1* locus and PEX, the risk allele reversal in different populations is not well understood, reflecting the complex genetic component of the disease and the need for further studies. LOXL1 expression levels vary over the course of disease in individuals with PEX, with elevated expression early in disease and reduced expression later (Schlotzer-Schrehardt et al., 2008). Because LOXL1 levels appear lower and elastosis higher in advanced PEX, *Loxl1* knockout mice have been used as models to study pathology. In the present study, we characterized ocular and non-ocular elastosis phenotypes associated with *Loxl1^−/−^* mice with three different genetic backgrounds: C57BL/6 (BL/6), 129S×C57BL/6 (50/50) and 129S. Our major finding was that, even at a young age, the genetic background remarkably influences the elastosis phenotypes at both the systemic and ocular levels, with the BL/6 content having the greatest influence on the severity of elastosis. Ocular and non-ocular phenotypes are summarized in Table 1.

**Table 1.**
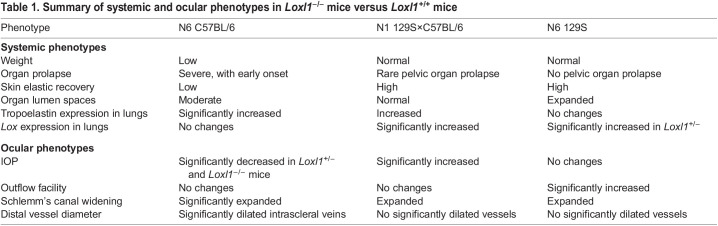
Summary of systemic and ocular phenotypes in *Loxl1^−/−^* mice versus *Loxl1*^+/+^ mice

Distinctively, BL/6 *Loxl1* null mice presented severe organ prolapse with an unexpectedly early onset. Our results showed that, in contrast with the other two backgrounds studied (50/50 and 129S), BL/6 *Loxl1^−/−^* mice experienced spontaneous rectal prolapse at 2 months of age with a high probability, accompanied by a significant weight loss. When *Loxl1* null mice were first generated in 129S×C57BL/6 chimeras, Liu et al. reported that these animals had mild rectal prolapse and females presented pelvic organ prolapse 1-2 days postpartum, with prolapse retraction over time but permanent pelvic tissue damage (Liu et al., 2004, 2006). The prevalence of anal prolapse in this mixed strain of null mice significantly increased past 8 months of age (Li et al., 2020). The unexpected high probability of prolapse at an early age in BL/6 *Loxl1^−/−^* mice limited our comparative study here to 2-month-old mice from all three genetic backgrounds. Importantly, the link between LOXL1 deficiency and pelvic organ prolapse reported in mice led to a study on the risk of PEX in women with pelvic organ prolapse, which found that diagnosis of PEX was increased by 1.56-fold in women with pelvic organ prolapse compared to that in unaffected individuals (Wirostko et al., 2016).

BL/6 mice also presented with increase skin laxity, reflected by reduced elastic recovery regardless of the genotype. Similar changes were reported by Liu et al. (2004) in ∼5-month-old *Loxl1^−/−^* mice on a mixed-strain background, although they only performed qualitative observations by scruffing their necks. LOXL1 is crucial for the formation, maintenance and remodeling of elastic tissue, acting not only as a crosslinking enzyme but also as a scaffolding element, and its function is not redundant with other members of the LOXL family (Liu et al., 2004; Csiszar, 2001). Thus, elastin-rich tissues, such as skin, lungs, blood vessels and ocular tissues, are severely affected by reduced levels of LOXL1 (Greene et al., 2020; Martínez-González et al., 2019). Although visual inspection of lung tissue sections stained with Weigert's Resorcin-Fuchsin stain showed that alveolar spaces were moderately expanded in 2-month-old *Loxl1^−/−^* mice from the three different studied backgrounds, quantification of this morphological change showed that alveoli were significantly enlarged in 129S *Loxl1^−/−^* mice, but BL/6 and 50/50 *Loxl1^−/−^* mice showed only an increased trend. Compared to our 2-month-old mice, Bellaye et al. (2018) showed that 5-month-old BL/6N(Cg) *Loxl1^−/−^* mice have significantly enlarged alveolar spaces consistent with emphysematous changes, compared to those of *Loxl1^+/+^* littermates. However, the mice in the study by Bellaye et al. (2018) were of a different genetic background and age, and the method used for quantification differed from the one used here. Surprisingly, in our study, only 129S *Loxl1* null mice showed significant enlargement in the alveolar spaces but did not show any other elastosis characteristics at an early age. Thus, 129S *Loxl1^−/−^* mice were not prolapsing and they did not present changes in weight, skin laxity or tropoelastin expression levels in lung tissue homogenates, suggesting that the 129S background is protective against *Loxl1*-induced elastosis. It is also strange that lung tissues from 129S *Loxl1^−/−^* mice did not have elevated levels of tropoelastin expression. Elastin consists of tropoelastin units covalently crosslinked to form long-lasting fibers (Lockhart-Cairns et al., 2020), and increased deposition of tropoelastin has been related not only to PEX (Bernstein et al., 2019; Zenkel and Schlötzer-Schrehardt, 2014a,b), but also to many other diseases (Heinz, 2021). Instead, tropoelastin expression was only significantly augmented in lung tissue lysates from BL/6 *Loxl1^−/−^* mice. Increased tropoelastin deposition was previously reported by others not only in lungs, but also in the cervix, uterus, skin, aorta, colon, bladder and anterior chamber tissues in older mice from the mixed strain (Liu et al., 2004; Wiggs et al., 2014).

About half of the individuals with PEX develop PEXG, with significant threat to vision (Ritch, 1994; Ritch, 2018; Grzybowski et al., 2019). In these individuals, LOXL1 expression levels change during the progression of the disease, increasing in early stages and decreasing in advanced stages of PEX and PEXG (Schlotzer-Schrehardt et al., 2008). Additionally, IOP is elevated due to increased outflow resistance in PEXG (Johnson and Brubaker, 1982; Gharagozloo et al., 1992). In our study, we did not observe the usual relationship between outflow facility and IOP, suggesting that confounding variables affect IOP. For example, IOP was significantly decreased in *Loxl1^+/−^* and *Loxl1^−/−^* mice on the BL/6 background compared to that in *Loxl1^+/+^* littermates, but outflow facility was not different between genotypes in the BL/6 mice. With the 50/50 mice, IOP was significantly increased and outflow facility was not different in both *Loxl1^+/−^* and *Loxl1^−/−^* mice compared to *Loxl1^+/+^* littermates. Curiously, IOP was unchanged in *Loxl1^−/−^* mice on the 129S background, but outflow facility was elevated. High IOPs with high outflow facilities in older *Loxl1^−/−^* mice were attributed to a significantly elevated episcleral venous pressure, supported by other findings such as dilation of the distal veins and an enlarged SC lumen containing erythrocytes. This made sense as the vasculature, particularly veins, is susceptible to LOXL1 deficiency due to reliance on elastin instead of collagen for structural support (Heinz, 2021; Schmelzer and Duca, 2022; Wang et al., 2021). Although our 2-month-old 50/50 *Loxl1* null mice showed a trend towards increased SC lumen diameter, we observed no changes in distal vessels at this young age. We note that true sizes of SC and distal vessels may have been distorted because eyes were fixed by immersion rather than perfusion, although relative differences between groups are comparable as all eyes were handled identically. PEX is primarily a condition associated with aging and LOXL1 expression levels are altered with time (Pasutto et al., 2017; Schlotzer-Schrehardt and Naumann, 2006; Schlötzer-Schrehardt, 2011; Behmoaras et al., 2008) Most of the studies using *Loxl1* null mice previously were carried out in mice older than 5 months, as it is known that elastosis progresses with age. However, our study was limited by severe prolapse in young *Loxl1* null BL/6 mice, constraining comparative experiments to 2-month-old animals. Even so, young BL/6 mice clearly demonstrated an elastosis phenotype, particularly in tissues such as the rectum that are subjected to repetitive mechanical stress. As such, a meta-analysis of PEX and PEXG patients identified only 12 patients between the ages of 13 and 40 years worldwide, all discovered as consequences of disruption of blood-aqueous barrier function due to intraocular surgery for other glaucoma conditions, ocular diseases or ocular trauma (Mayro et al., 2021). Although young BL/6 mice showed the most dramatic signs of elastosis, there were indications of elastosis in young mice on the other two backgrounds. For example, the 50/50 *Loxl1* null mice presented with several early signs of elastosis that were closely related to the well-studied original mixed strain. There were signs of increased skin laxity, dilation of SC and distal veins and increased alveolar size. 129S *Loxl1* null mice appeared to be resistant to manifesting elastosis characteristics at a young age, except for significantly enlarged alveolar spaces in lungs. These contrasting findings between 129S and BL/6 mice were observed previously in mice carrying a *Lmx1b* mutant allele on the BL/6 background, which were prone to develop abnormal IOPs, severe anterior segment developmental anomalies and optic nerve damage compatible with glaucoma, whereas 129S mice were resistant to developing these phenotypes (Tolman et al., 2021). We further looked at the expression of other lysyl oxidases in lung tissue to elucidate whether the puzzling changes reported in the young *Loxl1^−/−^* mice were due to potential compensatory mechanisms. Even though others have reported that LOXL1 function is nonredundant (Csiszar, 2001; Liu et al., 2004), we found that there was an inconsistent indication of compensation with upregulation of Lox mRNA levels in 50/50 and 129S mice, but not in BL/6 mice. It is important to note that LOX and LOXL1 are structurally and functionally more closely related than the other lysyl oxidase family members (Greene et al., 2020).

In summary, we have shown that *Loxl1* deficiency in young BL/6 mice severely affects elastic tissues, elastosis worsens with age in mixed or 50/50 animals, whereas the 129S background appears protective against elastosis. This indicates that there are other genes that are responsible for susceptibility or resistance to the development of pathological elastosis. Future studies on determining those loci associated with the elastosis phenotype in the *Loxl1^−/−^* mouse model are necessary. In this study, there were contradictory findings that may have been resolved if we were able to study older mice. Regardless, as a whole, these findings strongly implicate genetic context in susceptibility to *Loxl1^−/−^*-induced elastosis and direct future studies at identifying genetic modifiers that contribute to phenotypic severity of PEX in human patients.

## MATERIALS AND METHODS

### Mice

For this study, mixed 129S×C57BL/6.*Loxl1^+/−^* mice (Li et al., 2020) were backcrossed onto the C57BL/6 or 129S1/SvImJ background. C57BL/6J and 129S1/SvImJ mice were purchased from the Jackson Laboratory (Bar Harbor, ME, USA) (Fig. S1). Mice were bred/housed in clear cages and kept in housing rooms at 21°C on a 12 h:12 h light:dark cycle. Water and food were available *ad libitum*. *Loxl1^+/−^* mice from the different backgrounds were inbred to generate *Loxl1^+/+^*, *Loxl1^+/−^* and *Loxl1^−/−^* mice. Early onset and high prevalence of spontaneous anal prolapse in BL/6 background mice required backcrosses to stop at generation 6 (N6) and study of younger animals. We confirmed at least 98% C57BL/6 background at N6 using DartMouse^TM^ genotyping services (Geisel School of Medicine at Dartmouth) (Fig. S2). Thus, only 2-month-old (male and female) N6 C57BL/6 (BL/6), N1 129S×C57BL/6 (50/50) and N6 129S1/SvImJ (129S) *Loxl1^+/+^*,*Loxl1^+/−^* and *Loxl1^−/−^* mice were compared in this study. The number of animals used is denoted in the figure legends (*n*). When not referring to the number of mice, it is stated specifically. No randomization technique or software was used to allocate animals to experimental groups. Groups were determined solely by the genotype of the animal. Genotyping for the identification of *Loxl1^+/+^*, *Loxl1^+/−^* and *Loxl1^−/−^* mice was performed using the Terra^TM^ PCR Direct Genotyping kit following the manufacturer’s protocol (Takara Bio USA). Primers were designed for wild-type (WT) exon 1 (345 bp product) and the inserted mutant Neo cassette that introduces a translational stop codon in exon 1 of the *Loxl1* transcript (∼300 bp product) (WT forward primer, 5′-CGGACCTACGAACAGGGCTACG-3′; mutant forward primer, 5′-GAGATCAGCAGCCTCTGTTCCAC-3′; and WT/mutant reverse primer, 5′-ACACGTCGGTGCTGGGATCA-3′) (Integrated DNA Technologies) (Li et al., 2020). Animal procedures were in accordance with the animal care and use guidelines of Duke University (IACUC animal protocol A206-20-10) and in compliance with the Association for Research in Vision and Ophthalmology (ARVO) Statement for the Use of Animals in Ophthalmic and Vision Research.

### IOP measurements

Mice were weighed and then anesthetized using the SomnoSuite System (Kent Scientific Corporation). Briefly, mice were placed into the induction chamber, isoflurane was set at 2% and the flow rate was set at 400-600 ml/min. Upon cessation of movement, corroborated with lack of response to a toe pinch, mice were moved to a custom, heated immobilization platform and anesthesia was administered through a nose cone (settings for isoflurane: 1% isoflurane, 30-40 ml/minute). Using a stereomicroscope to position a stabilized tonometer probe in the center of the cornea and foot pedal switch to activate the tonometer (Wilson et al., 2022), IOP was measured within 5 min of anesthesia induction between 14:00 and 16:00 (TonoLab, Icare, Raleigh, NC, USA). Each recorded IOP value was the average of six measurements, giving a total of 36 rebounds from the same eye per recorded IOP value. Each measurement was the average of four single readings, automatically excluding the highest and lowest results (https://tonovet.com/wp-content/uploads/2015/12/TONOLAB_quick_guide_lo-res.pdf).

### Cutometer readings

Skin elasticity was determined using a cutometer (Cutometer dual MPA 580, Köln, Germany) (Abbas et al., 2022; Ohshima et al., 2013; Hara and Verkman, 2003) immediately after mice were euthanized. Measurements were performed on approximately 1 cm^2^ of shaved skin in the abdominal area, below the sternum. The probe was placed perpendicular to the skin and the elasticity was measured for 20 s using mode 1, and a total of four curves per mouse were collected. Skin elastic recovery (Q0) data were used to analyze responses.

### Outflow facility measurements

Outflow facility measurements were carried out on whole eyes *ex vivo* using the iPerfusion system (Sherwood et al., 2016; De Ieso et al., 2020; Li et al., 2020). Mice were euthanized using isoflurane and eyes were carefully enucleated. Paired eyes were mounted on a stabilization platform in perfusion chambers using a small amount of cyanoacrylate glue (Loctite, Westlake, OH, USA). The perfusion chambers were filled with prewarmed D-glucose in PBS (DBG, 5.5 mM) and temperature was maintained at 35°C. A pulled, beveled and sharpened glass microneedle was inserted into each anterior chamber using a micromanipulator while being visualized under a stereomicroscope. Both eyes were perfused with degassed, filtered DBG at 12 mmHg for 30 min for acclimatization, followed by nine sequential pressure steps starting at 5 mmHg, increasing by 1.5 mmHg until the pressure reached 17 mmHg, then decreasing to 8 mmHg for the last step. Data analysis was performed using a nonlinear flow-pressure model to account for the pressure dependence of outflow facility in mice, and the order (left versus right) in which eye perfusions were performed was randomized. The perfusionist was masked to the genotype of the eyes.

### Histochemistry and transmission electron microscopy

Mouse eyes were enucleated and immediately fixed in 4% paraformaldehyde at 4°C overnight. After removal of the posterior segments and lenses, anterior segments were embedded in paraffin or were cut into four quadrants and embedded in Epon. Left lungs were also collected, fixed in 4% paraformaldehyde at 4°C overnight, and embedded in paraffin. For elastin-collagen staining, 10 µm sections were deparaffinized and stained with Weigert's Resorcin-Fuchsin stain and Van Gieson's stain with Hematoxylin nuclear stain (26370-01 to -05, Electron Microscopy Sciences, Hatfield, PA, USA) and examined by light microscopy (Axioplan2, Carl Zeiss MicroImaging, Thornwood, NY, USA). Semithin sections (0.5 µm) from the Epon-embedded quadrants were cut using an ultramicrotome (LEICA EM UC6, Leica Mikrosysteme, Wien, Austria) stained with 1% Methylene Blue and visualized by light microscopy (Axioplan2). For electron microscopy studies of the outflow tissue ultrastructure, Epon-embedded quadrants were cut in 65 nm ultrathin sagittal sections using the previously mentioned ultramicrotome. Sections were stained with uranyl acetate/lead citrate and examined using a JEM-1400 electron microscope (JEOL USA, Peabody, MA, USA) at 5000×, 8000× and 15,000× magnification.

### Alveolar space measurements in lung sections

Alveolar spaces measurements were generated using the particle analysis function in Fiji. Briefly, lung sections were imaged at 20× magnification (Weigert's Resorcin-Fuchsin stain) and five random images from each genetic background (BL/6, 50/50 and 129S) and each genotype (*Loxl1^+/+^*, *Loxl1^+/−^* and *Loxl1^−/−^*) were used for quantification. Masked images were then opened in Fiji, converted to 8-bit grayscale, and an appropriate threshold was set to convert the image to black and white. Criteria for particle measurements were set: size – 25-Infinity (μm^2^); circularity – 0.00-1.00; output – show overlay masks. The resulting analyzed image was compared to the original image and non-alveolar spaces were excluded (see Fig. S3). Generated measurements were used to calculate the average overall area of the image, as well as the average particle size.

### Immunofluorescence and confocal microscopy for vessel measurements

Enucleated eyes were fixed in 4% paraformaldehyde at 4°C overnight. Anterior segments were cut posterior to the limbus, the lenses were removed and four centripetal relief cuts were made to facilitate mounting of the whole anterior segments onto a slide, prior to immunofluorescence studies. The anterior segments were permeabilized with 1% Triton X-100 in PBS, blocked with 5% goat serum in 0.1% Triton X-100 in PBS for 1 h at room temperature on a rocker, and then incubated with the primary antibody Armenian Hamster monoclonal anti-CD31 (PECAM1) (clone 2H8; 1:100; Developmental Studies Hybridoma Bank, University of Iowa) at 4°C overnight on a rocker. The primary antibody solution was removed and the tissue washed three times with 0.1% Triton X-100 in PBS (20 min), wholemounts were incubated with the appropriate fluorophore-conjugated secondary antibody diluted in blocking buffer (1:500; 127-605-160, Jackson ImmunoResearch Laboratories, Carlsbad, CA, USA) at 4°C overnight. After another round of washing (three times for 20 min) with 0.1% Triton X-100 in PBS, whole anterior segments were flat-mounted in Immuno-Mount^TM^ (Thermo Fisher Scientific) and imaging was performed at 20× using a Nikon Eclipse Ti2 inverted confocal microscope (Nikon, Melville, NY, USA). Vessel diameters were measured using ImageJ by applying a grid and measuring vessel widths at each point where a grid line crossed the vessel. Individual vessel measurements were averaged to give an average vessel width per wholemount image. (Li et al., 2020; Kizhatil et al., 2014; De Ieso et al., 2020).

### RNA isolation and cDNA synthesis

Right lungs were collected, split in two samples (for RNA isolation and immunoblotting), flash frozen and stored at −80°C until processing. Tissues were trimmed and dissociated using a pestle, and RNA isolation was performed using the RNeasy mini kit (74104, Qiagen) according to the manufacturer's protocol. After RNA isolation, cDNA synthesis was performed using 1 µg of isolated RNA per sample and cDNA was generated using the High-Capacity cDNA Reverse Transcription Kit (4368814, Applied Biosystems).

### qPCR

qPCR was performed using TaqMan Fast Advanced Master Mix (4444557, Thermo Fisher Scientific), TaqMan assays (Mm00495386_m1: *Lox*; Mm00804740_m1: *Loxl2*; Mm01184865_m1: *Loxl3*; Mm00446385_m1: *Loxl4*; Mm99999915_g1: *Gapdh*; Thermo Fisher Scientific) and QuantStudio3 (Applied Biosystems, Waltham, MA, USA) as per the manufacturer’s protocol.

### Immunoblotting

Right lungs were collected, flash frozen and stored at −80°C until processing. For protein isolation, tissue was homogenized and resuspended in RIPA buffer (89900, Thermo Fisher Scientific) containing 1% protease inhibitor (Sigma-Aldrich, St. Louis, MO, USA). Samples were placed on ice for 30 min, spun down at 14,000 ***g*** for 15 min at 4°C and the supernatant was transferred to a new tube. Protein concentration was determined using the Pierce BCA Protein Assay kit (23225, Thermo Fisher Scientific) and the colorimetric reaction was detected using a plate reader (SpectraMax M5 plate reader, Molecular Devices, San Jose, CA, USA).

For immunoblotting, samples were diluted in 4× Laemmli Sample Buffer (Bio-Rad Laboratories, Hercules, CA, USA), β-mercaptoethanol (Sigma-Aldrich) and urea (Sigma-Aldrich) and incubated at room temperature for 10 min. Diluted samples containing 10 μg of protein were loaded onto a 10% SDS gel. After electrophoresis, proteins were transferred to nitrocellulose membranes (Bio-Rad Laboratories) using the wet-transfer method and blocked for 1 h in 5% BSA and 0.1% Tween 20 in TBS at room temperature. Western blotting was used to detect elastin (1:1000, ab217356, Abcam) and GAPDH (1:3000, 2118, Cell Signaling Technology, Danvers, MA, USA) was used as the loading control protein. Membranes were developed with ECL Select or ECL Prime (Cytiva, Marlborough, MA, USA) and imaged using the ChemiDoc Touch Imaging System (Bio-Rad Laboratories). Densitometry measurements were performed with Image Lab 5.2.1 software (Bio-Rad Laboratories).

### Statistical analysis

The sample size was defined using an online calculator (https://sample-size.net/sample-size-means/) with alpha set to 0.05, power set to 0.8, proportion of subjects allocated to each group set to 0.5, effect size set to 0.5, and standard deviation of the outcome in the population set to 0.5. Using the T statistic and non-centrality parameter, the minimum *n* is 4. Using the Z statistic, the minimum *n* is 3. The Z statistic approximates the T statistic but gives sample sizes that are smaller. Therefore, we opted to aim for a sample size of at least three for each study group (Cohen, 1988; Smith, 2004). All samples and animals were included in the analysis as long as the technical quality of the data was preserved. Results are presented as mean±s.d. and significance was set at *P*<0.05. Statistical analyses were performed using two-tailed unpaired Student's *t*-test or one-way ANOVA test with Tukey's multiple comparison test as the post hoc test. GraphPad Prism 9.5.0 software was used to perform all the analyses.

## Supplementary Material

10.1242/dmm.050392_sup1 Supplementary information
