# Supplementary material for: Genetic background determines severity of Loxl1-mediated systemic and ocular elastosis in mice
Source: Dis Model Mech. 2023 Nov 13;16(11):dmm050392. doi: 10.1242/dmm.050392 (PMC10668029; doi:10.1242/dmm.050392)
Supplement: Supplementary information [file dmm-16-050392-s1.pdf]

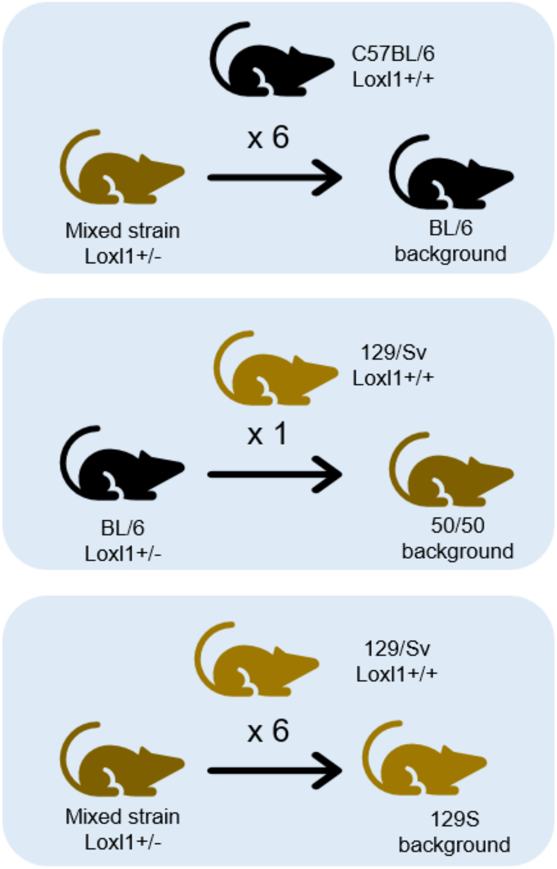

Fig. S1. Shown is the scheme used for mouse backcrossing. Mixed 129S.C57BL/6.*Loxl1*$^{+/-}$ mice were backcrossed onto C57BL/6J or 129S1/SvImJ background six times to obtain the BL/6 (upper panel) or 129S (lower panel) background mice. BL/6 mice were backcrossed one time with 129S1/SvImJ, producing the 50/50 strain (middle panel)

Disease Models & Mechanisms • Supplementary information

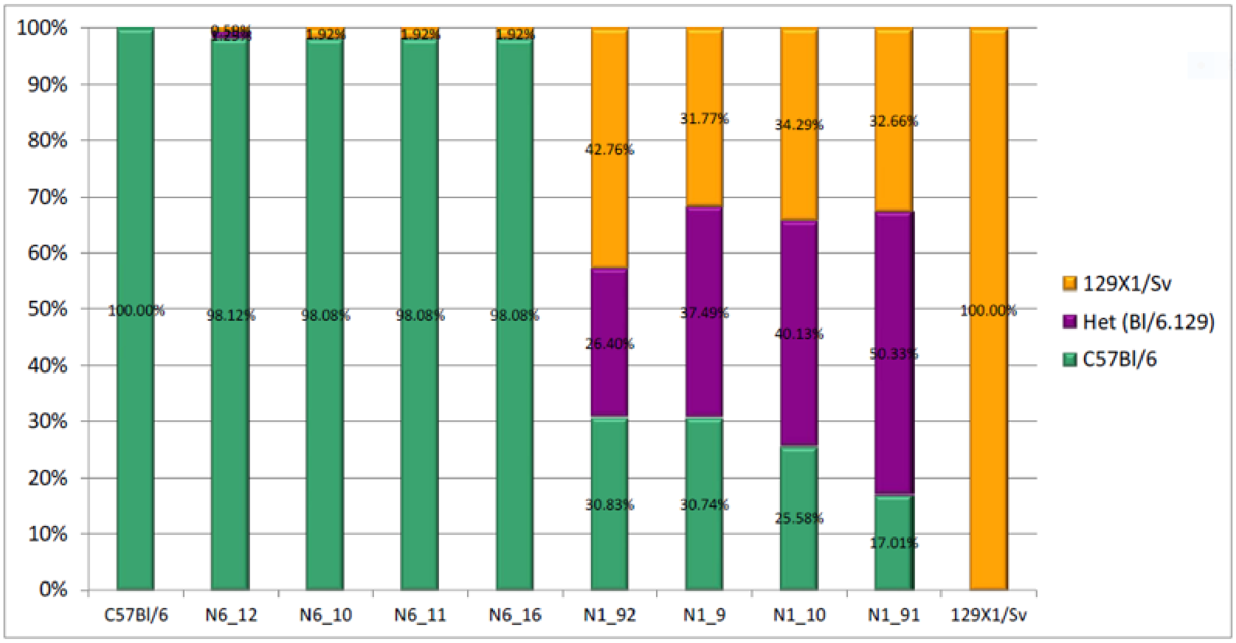

Fig. S2. Full genotyping of backcrossed BL/6 and 50/50 *Loxl1*$^{+/+,\ +/-,\ -/-}$ mice using DartMouseTM genotyping services (Dartmouth- Geisel School of Medicine).

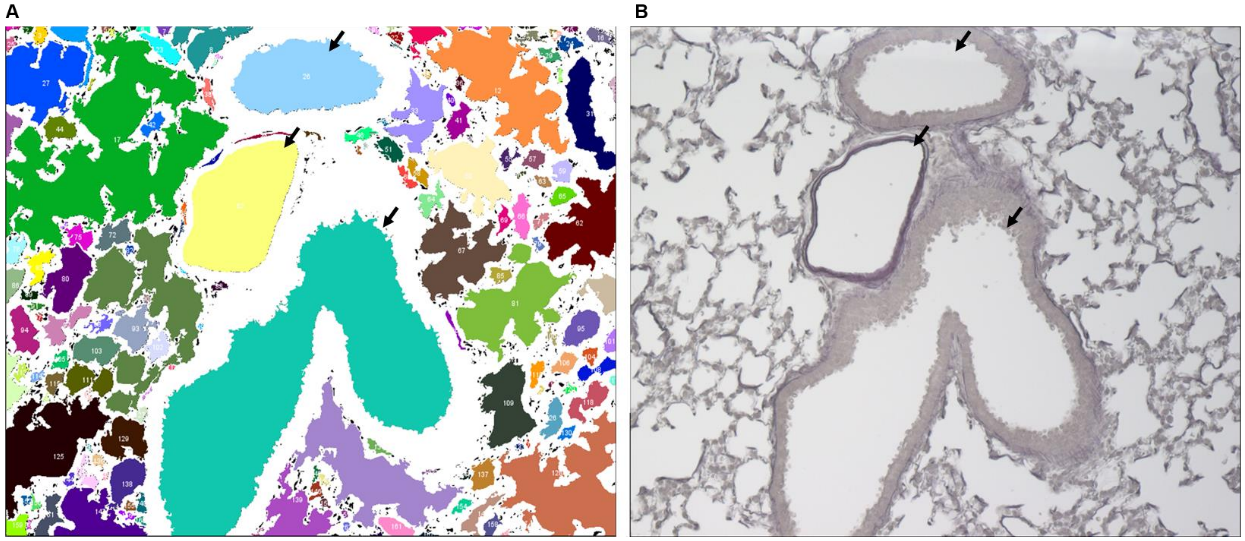

Fig. S3. Representative image showing lung alveolar space quantification using particle analysis in Fiji. (A) Each measured space/result has an assigned number (and color) by Fiji. (B) Using the original image as a guide, non-alveolar spaces are excluded from the analysis. (arrows point major vessels that were excluded) (image from a 50/50.*Loxl1*$^{-/-}$ mouse).

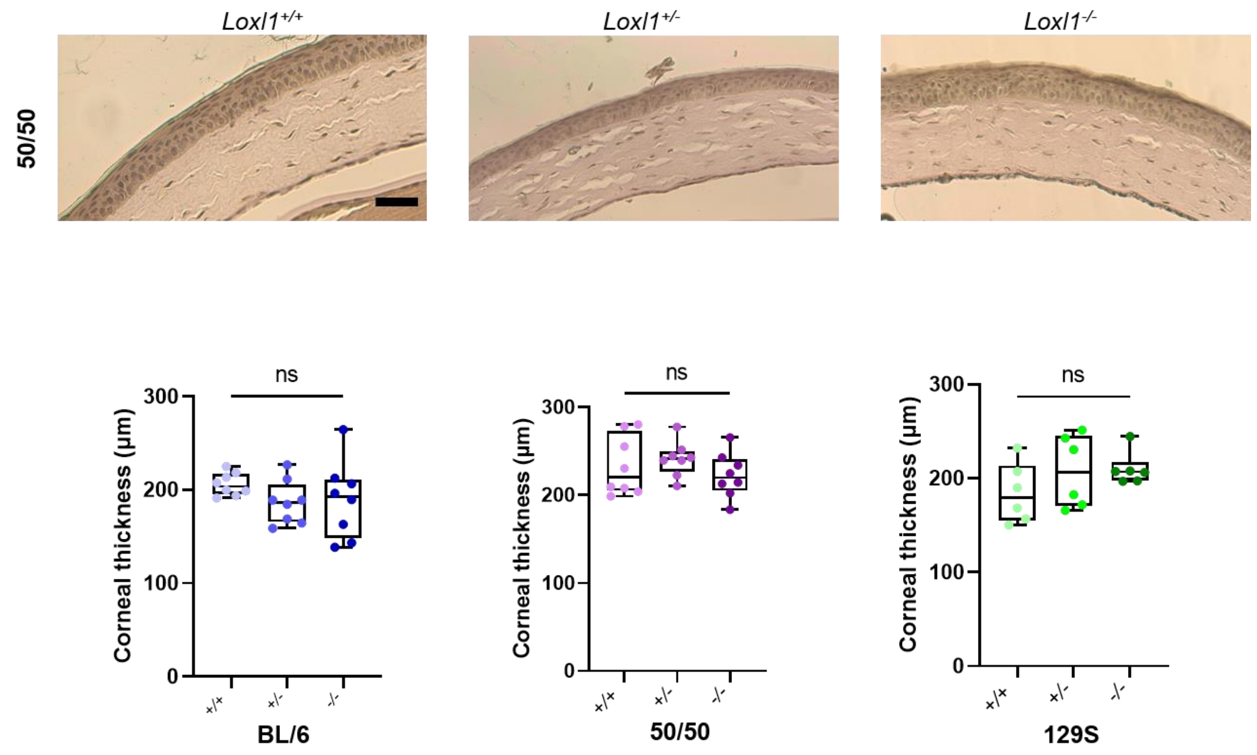

Fig. S4. Central corneal thickness is not altered in *Loxl1* deficient mice. Upper panel shows representative images from sagittal sections of corneas stained with *Weigert's Resorcin-Fuchsin* stain in 50/50 *Loxl1*$^{+/+}$, *Loxl1*$^{+/-}$ and *Loxl1*$^{-/-}$ mice. Lower panel: corneal thickness quantification did not show significant changes in the *Loxl1*$^{-/-}$ mice when compared to the respective *Loxl1*$^{+/+}$ littermates. Measurements were performed on the central cornea, using the pupil area as a guide (iris' border) (n= 2 sections /3-4 eyes/ mice/genotype/background). Scale bar = 50 µm. Ordinary one-way ANOVA followed by Tukey's multiple comparisons test was used for statistical analysis.

Disease Models & Mechanisms • Supplementary information

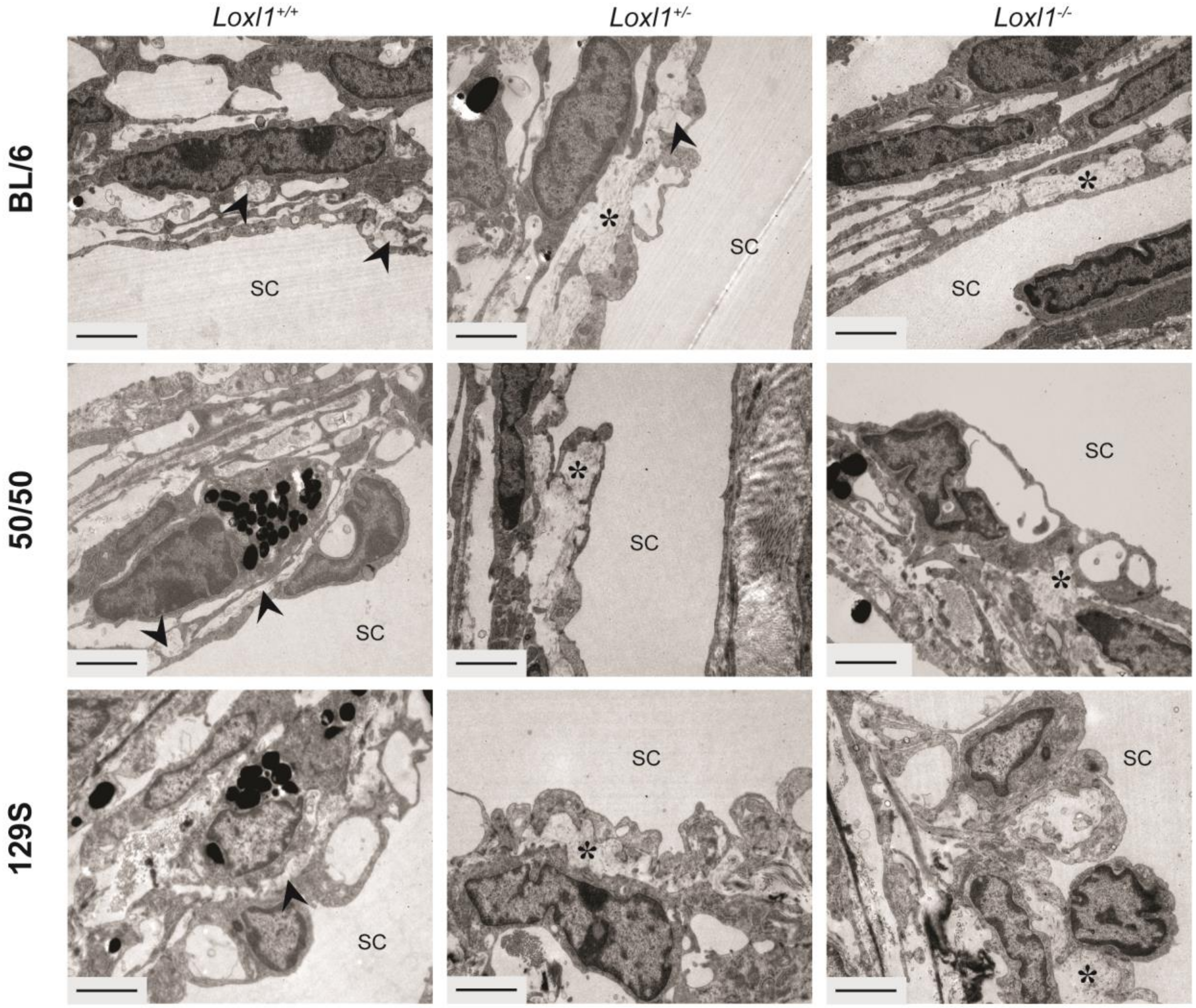

Fig. S5. Young *Loxl1* deficient mice displayed irregular extracellular matrix deposition below the inner wall of Schlemm's canal. Representative anterior segment ultrathin section images for *Loxl1*$^{+/+}$, *Loxl1*$^{+/-}$ and *Loxl1*$^{-/-}$ mice from BL/6, 50/50 and 129S background showing discontinuous basal laminar material in *Loxl1*$^{+/+}$ mice (arrowheads) and more continuous and abound material in *Loxl1*$^{+/-}$, increasing even more in *Loxl1*$^{-/-}$ mice (*). SC=Schlemm's canal. (BL/6, 50/50 and 129S, n= 3-5 sections per eye / 4 eyes /genotype). Scale bar = 2 µm.

Disease Models & Mechanisms • Supplementary information
